# Chromatin Landscape During Skeletal Muscle Differentiation

**DOI:** 10.3389/fgene.2020.578712

**Published:** 2020-09-18

**Authors:** Oscar Hernández-Hernández, Rodolfo Daniel Ávila-Avilés, J. Manuel Hernández-Hernández

**Affiliations:** ^1^Laboratory of Genomic Medicine, Department of Genetics, Instituto Nacional de Rehabilitación Luis Guillermo Ibarra Ibarra, Mexico City, Mexico; ^2^Laboratory of Epigenetics of Skeletal Muscle Regeneration, Department of Genetics and Molecular Biology, Centro de Investigación y de Estudios Avanzados del Instituto Politécnico Nacional (CINVESTAV), Mexico City, Mexico

**Keywords:** MyoD, myogenic regulatory factors, satellite cells, myogenesis, muscle regeneration

## Abstract

Cellular commitment and differentiation involve highly coordinated mechanisms by which tissue-specific genes are activated while others are repressed. These mechanisms rely on the activity of specific transcription factors, chromatin remodeling enzymes, and higher-order chromatin organization in order to modulate transcriptional regulation on multiple cellular contexts. Tissue-specific transcription factors are key mediators of cell fate specification with the ability to reprogram cell types into different lineages. A classic example of a master transcription factor is the muscle specific factor MyoD, which belongs to the family of myogenic regulatory factors (MRFs). MRFs regulate cell fate determination and terminal differentiation of the myogenic precursors in a multistep process that eventually culminate with formation of muscle fibers. This developmental progression involves the activation and proliferation of muscle stem cells, commitment, and cell cycle exit and fusion of mononucleated myoblast to generate myotubes and myofibers. Although the epigenetics of muscle regeneration has been extensively addressed and discussed over the recent years, the influence of higher-order chromatin organization in skeletal muscle regeneration is still a field of development. In this review, we will focus on the epigenetic mechanisms modulating muscle gene expression and on the incipient work that addresses three-dimensional genome architecture and its influence in cell fate determination and differentiation to achieve skeletal myogenesis. We will visit known alterations of genome organization mediated by chromosomal fusions giving rise to novel regulatory landscapes, enhancing oncogenic activation in muscle, such as alveolar rhabdomyosarcomas (ARMS).

## Introduction

### Skeletal Muscle Commitment and Differentiation: The Pioneer Factor Pax7

During development, progenitors are specified by the action of specific genes that establish the cellular fate of a plethora of cell lineages. Being the most abundant tissue in the vertebrate body, skeletal muscle plays a major role in physiological functions, such as locomotion, breathing, and energy metabolism (Morrison et al., 2008). In response to disease or injury, postnatal skeletal muscle has the remarkable ability to regenerate. This regenerative capacity of skeletal muscle relies on a subpopulation of cells, termed satellite cells that function as muscle stem cells. Satellite cells are marked by Pax3 and Pax7 expressions, regulators that belong to the Paired box DNA binding proteins. Pax proteins are divided into subgroups which Pax3 and Pax7 share due to their very similar roles in organ specification as well as similar binding targets (Seale et al., 2000; Kuang et al., 2006). Pax3 and Pax7 are two transcription factors essential for myogenesis as their ectopic expression alone is sufficient to induce a myogenic fate in mouse embryonic stem cell, and facilitate engraftment into muscle after transplantation (Darabi et al., 2011). Although Pax3 and Pax7 are co-expressed during embryonic development, in postnatal myogenesis, their function is significantly different (Kuang et al., 2006; Relaix et al., 2006). The most striking difference is that Pax7-null mice display severe characteristics such as a 50% reduction in weight compared to their wildtype counterparts and a reduction in muscle fiber size. Most importantly, Pax7-null mice do not possess satellite cells, leading to their death around the 2-week mark due to a lack of muscle regeneration and lack of functioning diaphragm (Seale et al., 2000). On the contrary, Pax3 was shown to be dispensable for the adult satellite cell function (Relaix et al., 2006). Pax7 is undoubtedly a master regulator of early myogenesis as its expression is essential for satellite cell and myoblast cell cycle progression and proliferation. Molecular differences on the function of Pax3 and Pax7 may be partially explained by their respective affinities for a DNA binding site. Indeed, by over-expressing TAP-tagged Pax3 and Pax7 constructs into mouse primary myoblasts, Soleimani et al. (2012a) generated a genome-wide Pax3 and Pax7 binding repertoire, where important differences arose. For instance, they reported that Pax7 binds nearly 52,600 sites, whereas Pax3 binds to 4,648 sites in the genome. In addition, they reported co-binding at 1,200 genomic sites. Mechanistically, these differences in the number of binding sites were attributed to the dominant ability of Pax7 over Pax3 to recognize the element – TAAT – at its cognate binding site, through its homeodomain (Soleimani et al., 2012a). Therefore, while Pax3 binds a subset of Pax7 target genes that are mainly involved in the regulation of embryonic functions and maintenance of an undifferentiated phenotype, Pax7 specifically activates genes involved in the maintenance of adult satellite cell phenotype, from the regulation of proliferation to inhibition of differentiation (Soleimani et al., 2012a; Hernández-Hernández et al., 2017). Despite this emerging genomic characterization of Pax7 and Pax3, there is no further biological insight into the mechanistic function of these two factors over chromatin organization in satellite cells or mouse embryo development. One important question to be addressed in future experiments would be the potential relationship between Pax3/Pax7 and MyoD during myogenesis. Would it be an overlap between Pax3/Pax7 and MyoD? If so, what would be the potential effects in terms of molecular hierarchy and progression of gene expression during differentiation of muscle progenitors?

A critical question related to gene transcription and cell reprogramming is how transcription factors gain access to their cognate DNA-binding motifs within condensed chromatin to activate lineage programs. Pioneer transcription factors are characterized by having the unique property of enabling the opening of closed chromatin sites, for implementation of genetic cell fates (Soufi et al., 2012). Pax7 has been reported to be a pioneer factor in the context of pituitary melanotrope development (Budry et al., 2012). Although Pax7 does not play a maintenance role in the pituitary, as it does in muscle satellite cells, melanotrope Pax7-positive cells are engaged in the differentiation pathway but need another fundamental component to complete the process, the T-box transcription factor Tpit (Mayran et al., 2019). Thus, Pax7 preferentially recognizes a motif composed of binding sites for its two DNA binding domains, the homeo and paired domains, recognizing its entire target sequence on nucleosomal DNA (Mayran et al., 2019). This leads to greater binding stability and allows for pioneer action. Then after Pax7 recognizes and engages pioneering sites, Tpit later provides the chromatin opening ability and melanotrope terminal differentiation through deployment of melanotrope-specific enhancer repertoire (Budry et al., 2012). Whether any assistant co-factor of Pax7 is needed in the case of satellite cells in order to induce gene expression is still unknown, partially due to limitations in the number of muscle stem cells available in the muscle tissue, leading to technical difficulties to address this unknown aspect of muscle stem cells function. A plausible strategy to identify new co-factors involved in the Pax7 regulatory networks of myogenesis would be the analysis of putative composite paired and homeo motifs derived from previous studies, such as that of Soleimani et al. (2012a).

### Molecular Determinants of Muscle Regeneration, MyoD as Master Epigenetic Regulator

**Figure 1:**
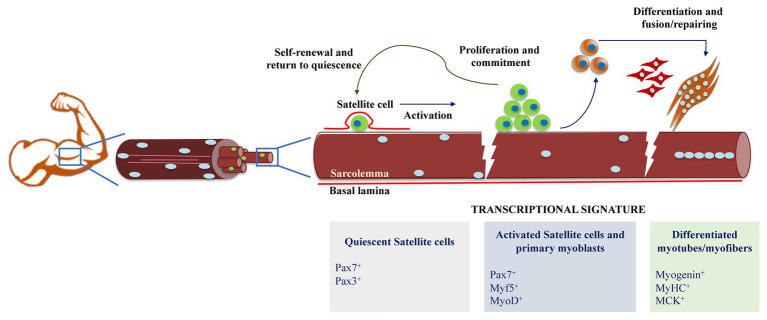
Schematic representation of skeletal muscle differentiation. Muscle regeneration is possible thanks to the functionality of adult muscle stem cells and the satellite cells. In homeostatic conditions, satellite cells are in a quiescent state, and after different stimulus caused by damage, they proliferate to generate myogenic precursors and to repopulate the satellite cell niche. Myoblasts express markers of muscle identity and fuse to each other to generate myotubes and myofibers, to eventually repair the damaged muscle fiber.

Highly regulated transcriptional gene regulatory networks hierarchically control myogenic differentiation, each under the precise control of a master regulator present at specific temporal and spatial developmental stages (Figure 1; Hernández-Hernández et al., 2017). The activation of the myogenic regulatory factor Myf5 marks the commitment of satellite cells to enter the pathway toward terminal differentiation. One of the best characterized genes regulated by Pax7 in muscle stem cells is Myf5. Binding of Pax7 to enhancer elements 57 and 111 kb upstream of the Myf5 transcription start site marks the recruitment of the Trithorax complex, which is composed of Ash2l, Wdr5, Rbbp5, and MLL1/2 to establish a permissive epigenetic state through trimethylation of histone 3 lysine 4 (H3K4me3; McKinnell et al., 2007; Soleimani et al., 2012a). An additional molecular switch to engage myogenic commitment in satellite cells is driven by the methylation of the amino-terminus domain of Pax7 by the action of the arginine methyltransferase Carm1. This results in the subsequent recruitment of MLL1/2 and the Trithorax complex composed of Ash2l and Wdr5 at the Myf5 locus (McKinnell et al., 2007; Kawabe et al., 2012). Importantly, the absence of Carm1 methylation activity in satellite cells is enough to dramatically reduce the regenerative potential of muscle stem cells. However, it is still unclear whether this dramatic effect is only due to the Carm1 action over Pax7 itself or a combined effect on global histone methyltransferase activity (Kawabe et al., 2012). While activating Myf5 expression, it has also been suggested that Pax7 may antagonize myogenic progression by repressing genes needed for muscle differentiation. Indeed, it has been reported that Pax7 over-expression is enough to downregulate MyoD expression (Olguin and Olwin, 2004; Zammit et al., 2006). However, there is limited mechanistic evidence of how Pax7 might induce expression of certain set of genes while keeping repressive signals over others.

Once the activation of terminal myogenic program is triggered, the progression of development and differentiation of muscle lineage is regulated by the family of the basic helix-loop-helix (bHLH) myogenic regulatory factors (MRFs), composed by MyoD, Myf5, myogenin, and MRF4 (Hernández-Hernández et al., 2017). Structurally, the four MRFs share a similar genomic organization, and the proteins have highly conserved 65 amino acid bHLH domains of which three specific residues encode myogenic specificity (Davis and Weintraub, 1992). The helix-loop-helix region allows dimerization with the E-proteins E12, E47, or HEB (Murre et al., 1989; Hu et al., 1992), while the basic domains of the heterodimers recognize E-box sites of the consensus sequence CANNTG enriched at gene regulatory elements of muscle specific genes (Rudnicki and Jaenisch, 1995). An additional conserved alpha-helical domain (helix III), located in the C-terminal portion of each MRF, is key to induce differentiation. The helix III on the C-terminal domain of MyoD is key for the interaction with the bHLH domain and for the recruitment of complexes with chromatin remodeling activity in order to allow the access to repressed loci through the N-terminal transactivation domain (Ishibashi et al., 2005). A classic example of this is represented by the factors Pbx/Meis, which have been observed constitutively bound at inactive and repressed myogenic MyoD target loci (Berkes and Tapscott, 2005). Through helix III, MyoD binds to Pbx/Meis, and this triggers the recruitment of complexes with histone acetyl-transferase activity. Interestingly this association not only culminates with acetylation of surrounding histones but also with the acetylation of MyoD (Dilworth et al., 2004; Berkes and Tapscott, 2005). Notably, by swapping experiments, it has been observed that the myogenin helix III acts more like a traditional activation domain and cannot substitute for that of MyoD in this sequence of molecular events, whereas the Myf5 and MRF4 helix IIIs are more similar to that of MyoD than that of myogenin (Bergstrom and Tapscott, 2001). In activated satellite cells, which do not express MRF4, this model therefore places Myf5 and MyoD in a key position upstream of myogenin in providing myogenic specification.

Despite their structural similarities, MRFs share limited functional redundancy; while a partial redundancy exists between Myf5 and MyoD (Braun et al., 1992; Rudnicki et al., 1992), the combined knock-out of both genes results in a complete absence of skeletal muscle (Rudnicki et al., 1993). In addition, muscle progenitors in the double-mutant MyoD^−/−^: Myf5^−/−^ mice acquire non-myogenic cell fates, indicative that either MyoD or Myf5 protein is required for muscle specification. In the single MyoD^−/−^ mice, myogenic cells compensate by upregulating Myf5 resulting in delayed differentiation, suggesting that Myf5 is initially insufficient for myogenic progression (Kablar et al., 1997). In contrast, while otherwise normal, Myf5^−/−^ mice display delayed myotome formation until MyoD activation (Braun et al., 1994). In addition, through genetic lineage tracing studies using Myf5nLacZ reporter mice, it was demonstrated that Myf5 is expressed in all embryonic muscles, indicating an essential role for this MRF in myogenic specification (Tajbakhsh et al., 1996).

Myogenin and MRF4 follow MyoD and Myf5 expressions in the muscle developmental program, and are required for myoblast fusion and terminal differentiation (Rudnicki and Jaenisch, 1995). While myogenin^−/−^ mice initiate myogenesis, they possess a perinatal lethal defect in terminal differentiation while retaining a normal number of undifferentiated mononuclear myoblasts (Hasty et al., 1993; Nabeshima et al., 1993). On the other side, while expressing higher levels of myogenin, MRF4^−/−^ mice develop normal muscle, suggesting a functional overlap (Zhang et al., 1995). This is further evident in MyoD^−/−^: MRF4^−/−^ mice, which display normal myogenin expression but phenocopy myogenin knock-out mice (Rawls et al., 1998). However, MRF4 may have a significant role in embryonic myogenesis with deficient mice exhibiting a range of phenotypes consistent with commitment, differentiation, and maintenance (Braun and Arnold, 1995; Kassar-Duchossoy et al., 2004). Cooperative function of additional coactivators during myogenesis includes the activity of MEF2 transcription factors (Molkentin and Olson, 1996). Indeed, it has been reported that all MRFs increase their transactivation activities when interacting with MEF2 (Buchberger et al., 1994; Black et al., 1995).

The early development of the C2 cell line (Yaffe and Saxel, 1977) as well as the cloning of the transcription factor MyoD (Lassar et al., 1986) were two initial contributions that set the foundations for our understanding behind muscle differentiation; being these abilities: (1) the development of a cell line model capable to form contractile myotubes *in vitro* and (2) the discovery of a factor whose introduction into many different lineages is able to induce a muscle cell phenotype (Buchberger et al., 1994; Black et al., 1995; Molkentin and Olson, 1996). Based on these observations, it is possible to include MyoD on the list of pioneering factors.

Classical studies were performed trying to explore the ability of MyoD to remodel chromatin from an inaccessible and repressed environment. From this, it was conclusive that only after MyoD expression, muscle-specific loci started to allow access to nucleases (Gerber et al., 1997). How this remodeling happens greatly depends on the recruitment of complexes with histone acetyl-transferase activities. Indeed, MyoD interacts with p300 and with the p300/CBP-associated factor (PCAF; Yaffe and Saxel, 1977; Puri et al., 1997a,b; Sartorelli et al., 1997), with the final outcome of not only direct histone acetylation but also acetylation of the MyoD DNA binding domain as well (Sartorelli et al., 1999; Dilworth et al., 2004). switching defective/sucrose non-fermenting (SWI/SNF) chromatin remodeling complex is also recruited by MyoD in a p38-MAPK-dependent manner (Simone et al., 2004). Conclusively, inhibition of either histone acetyl transferases activity or p38 activity leads to failure to initiate muscle specific loci activation (Serra et al., 2007). A MyoD dependent recruitment of SWI/SNF to target loci initially consist on the association with Brg1/Brm-associated factors (BAFs), which are alternatively incorporated into specific SWI/SNF complexes with patterns of tissue-specific expression (Wang et al., 1996). BAF60c followed by the core components Baf47, Baf155, and Baf170 are required for MyoD-initiated chromatin remodeling activity on myogenic loci (Forcales et al., 2011).

A requisite for myogenesis to occur is the removal of repressive marks surrounding chromatin at muscle promoters. Catalyzed and deposited by the activity of enhancer of zeste homolog 2 (Ezh2), the enzymatic subunit of the polycomb repressive complex 2 (PRC2), trimethylation of lysine 27 of histone 3 (H3K27me3) is one of the inhibiting signals for myogenic genes to be transcribed (Caretti et al., 2004; Hernández-Hernández et al., 2013). At the Pax7 promoter, Ezh2 is recruited during proliferation of committed myogenic cells. Upon treatment with anti-TNFα antibodies in dystrophic muscle, p38α mitogen activated protein kinase (MAPK) pathway resulted inhibited (Palacios et al., 2010). The authors show that in a regenerative context, inflammation-activated p38α promotes phosphorylation of Ezh2, which induces the formation of an Ezh2-transcription factor Ying Yang-1 (YY1) repressor complex at the Pax7 promoter. As mentioned earlier, as myoblasts progress on the differentiation program, Pax7 expression is downregulated. Participation of YY1 is also relevant for the spatial-temporal regulation of muscle genes. As a direct target of NF-κB, YY1 is expressed and recruited to genes activated at late times of differentiation, such as myosin heavy chain and muscle creatine kinase, in a complex with HDAC1 and Ezh2 (Wang et al., 2007). A mechanism by which YY1/Ezh2 repressor complexes are removed from muscle loci depends on the action of specific microRNAs (miRNAs). It has been shown that YY1 is a direct target of the miRNAs miR-34c, miR-29, and miR-1, leading to reduction of YY1 levels (Wang et al., 2008, 2017; Lu et al., 2012). This allows the deposition of an activator complex containing PCAF, SRF, and MyoD to induce transcription of muscle genes (Wang et al., 2007). Additional mechanism to reduce H3K27me3 marks is mediated by the demethylase UTX. For instance, at the enhancer element of myogenin and muscle creatine kinase genes, binding of the transcription factor Six4 initiates the recruitment of UTX with the concomitant reduction of H3K27me3. In addition, UTX spreads the activation signal into the coding region of the genes *via* a transcriptionally active RNA-Pol II mediated mechanism (Seenundun et al., 2010). The authors propose that Six4 is recruited by Mef2d, which in conjunction are able to recruit the demethylase UTX at muscle-specific genes.

During myogenesis, a specific set of genes is actively transcribed, such as those involved in specialized functions, whereas others need to be silenced; for instance, cell cycle regulation genes. Experimental evidence shows that MyoD has this dual activity in muscle differentiation by acting as a modular scaffold to assemble molecular switches to activate or repress gene expression (Tapscott, 2005). It has been shown that the activity of MyoD is impeded by the action of transcriptional repressors Snai1/2 through direct binding to E-boxes in undifferentiated myoblasts. Then Sna1/2 recruits HDAC1 to exclude MyoD from promoters and enhancers of muscle -specific loci (Soleimani et al., 2012b). As differentiation goes on, induction of miR30-a and miR206 negatively regulates Sna1/2 levels, leading to the replacement of Snai1/2-HDAC1 repressive complex for MyoD binding at E-boxes (Soleimani et al., 2012b). A similar mechanism was described in the case of the histone H3 lysine-9 specific methyltransferase, Suv39h1. Association of MyoD with Suv39h1 not only inhibits MyoD activity, but also spreads the repressive histone mark H3K9me3 at the myogenin promoter (Mal, 2006). In addition, HDAC1 is able to recruit Suv39h1 at MyoD regulated promoters to establish a repressor complex to control the spatial-temporal expression of muscle genes (Giacinti et al., 2006; Mal, 2006). How different classes of HDACs regulating myogenesis leave muscle promoters upon differentiation to allow muscle specific gene expression is dictated by several mechanisms. These include reduction in expression levels, nuclear export, or differential protein-protein interactions with co-activators or co-repressors. For example, HDAC1 interacts with MyoD in myoblasts at silenced muscle specific genes, whereas HDAC1expression is reduced as differentiation proceeds (Puri et al., 2001). A mechanism for the dissociation of the MyoD-HDAC1 complex is illustrated by the hypophosphorylation of the tumor suppressor pRb protein. In this scenario, multiple differentiation signals mimicked *in vitro* by serum removal, which induce pRb hypophosphorylation. As a consequence, pRb then recruits HDAC1, and this event allows the disassembling of the MyoD-HDAC1 complexes at muscle-specific regulatory elements and terminal differentiation (Puri et al., 2001). Additional signaling regulating the formation of repressive complexes is exemplified by the calcium/calmodulin-dependent protein kinase (CaMK; McKinsey et al., 2000; Zhang et al., 2002). As a promyogenic signal, CaMK phosphorylates HDAC4 and HDAC5, making them targets for nucleus exporting, and thus promoting the replacement of repressive complex with activating complex for muscle gene expression (McKinsey et al., 2000).

Critical events in the process of cell commitment and differentiation are regulated by the coordinated action of distal regulatory elements, typically enhancers that respond to tissue-specific transcription factors and co-activators (Heinz et al., 2015). Active enhancers are marked by H3K4me1, by the presence of histone acetyl transferases, relative enrichment in H3K27Ac, and by DNase hypersensitivity, which reflects chromatin accessibility (Visel et al., 2009; Krebs et al., 2011; Sartorelli and Puri, 2018). ChIP-seq analyses of H3K4me1, H3K27ac, p300, and RNA polymerase II in myoblasts and myotubes revealed that the total number of muscle loci with potential to be enhancer elements increased in a differentiation dependent manner. In undifferentiated cells, approximately 4,000 enhancers were predicted versus around 6,000 in myotubes. Nearly 3,000 of these putative enhancers were active only before differentiation, whereas 5,000 contained enhancer marks after induction of differentiation. An interesting observation was that the median enhancer-promoter distance for differentiated cells was shortened by 13 kb, compared with myoblasts, suggesting that changes in genomic distances could be an indicative of gene activation and muscle differentiation, and perhaps by the formation of higher-order chromatin contacts between distal regulatory elements and promoters. Interestingly, the overlap of these enhancer data sets with experimentally determined MyoD-binding events revealed that only approximately 30% of active enhancers were bound by MyoD (Blum et al., 2012; Blum and Dynlacht, 2013). In a subsequent study using C2C12 myoblasts and myotubes, Cao et al. (2010) performed a genome-wide analysis of MyoD binding during myogenic differentiation. They found that MyoD binds at a high number of DNA sites where no identifiable E-boxes at the binding sites. They found 23,000 and 26,000 MyoD binding sites in myoblasts and myotubes, respectively. In the vast majority of sites, MyoD binding was stable regardless the differentiation status, which was reviewed in Hernández-Hernández et al. (2017). It is worth to mention that the functionality as putative regulatory elements of most of these sites remain unexplored. In a more recent study, Mousavi et al. (2013) found nearly 39,000 sites bound by MyoD in C2C12 myotubes and close to 18,000 in C2C12 myoblasts. An interesting aspect of this work was the use of RNA-seq to show that the important fractions of the MyoD binding sites are bound by RNA polymerase II, are marked by H3K4me1and H3K27Ac, and are also actively transcribed in both senses in myoblasts and myotubes in the form of enhancer RNAs (eRNAs; Mousavi et al., 2013).

MyoD locus contains two main distal regulatory elements whose transcripts were detected in myotubes, one located approximately 20 kb from the MyoD promoter, called core enhancer (CE), and a distal regulatory region (DRR) at 5 kb upstream of MyoD transcriptional start site (Asakura et al., 1995; Chen et al., 2001; L’honore et al., 2003; Chen and Goldhamer, 2004; Gonçalves and Armand, 2017). Mousavi et al. (2013) determined that the CE-derived eRNA is recruited to the MyoD promoter region, suggesting a mechanism of regulation in *-cis*. This was confirmed by the use of small interfering RNAs (siRNAs)-based strategies to inhibit expression of these eRNAs. Interestingly, ablation of the MyoD-DRR did not affect MyoD expression but dramatically reduced mRNA levels of myogenin, whose gene is located at a different chromosome. On the contrary, overexpression of a DNA construct corresponding to MyoD-DRR was enough to induce myogenin expression. This argues in favor of a mechanism of regulation in *-trans* mediated by eRNAs in muscle differentiation (Mousavi et al., 2013). In a subsequent study, Tsai et al. (2018) used chromatin isolation by RNA purification sequencing (ChIRPseq; Chu et al., 2011) and single-molecule RNA fluorescent *in-situ* hybridization (smRNAFISH; Femino et al., 1998) to further confirm the binding of the eRNA MyoD-DRR at the myogenin locus. Furthermore, they demonstrated that MyoD-DRR binds to SCM, the core subunit of the cohesin complex and interacts with proteins important for biogenesis of eRNAs, such as WDR82 and members of the integrator complex (INT; Austenaa et al., 2015; Lai et al., 2015). Upon DRR-eRNA depletion, cohesin occupancy at myogenin promoter is reduced along with its mRNA levels. Interestingly, the authors did not find evidence of physical proximity between DRR enhancer regions of MyoD with myogenin promoter, making unfeasible the existence of a looping-mediated mechanism of myogenin expression under these experimental conditions (Tsai et al., 2018). A mechanism of how trans-acting eRNAs identify their cognate targets remains elusive; the authors proposed that the eRNAs polyadenylation signal may afford enough stability to explore the nuclear space and identify target sequences on which to act. For example, compared to the half time of 7 min observed for some eRNAs (Santa et al., 2010; Schaukowitch et al., 2014), the DRR-eRNA has a half-life of 30 min (Tsai et al., 2018), which may provide enough time to be directed toward its genomic target in the nucleus.

Growing body of evidences suggests a possible participation of MyoD in regulating the three-dimensional organization of chromatin during muscle differentiation. A first evidence emerged by demonstrating a physical and functional interaction between MyoD and the CCCTC-binding factor (CTCF) that results in activation of muscle-specific genes (Delgado-Olguín et al., 2011). In fact, CTCF depletion by morpholinos lead to somite disorganization in zebrafish, along with reduced muscle fibers and overall decrease in expression levels of muscle-specific markers (Delgado-Olguín et al., 2011). This shows that CTCF could act as a mediator necessary for transactivation of MyoD target genes and overall in myogenic differentiation. A second evidence is the observation that MyoD binding corresponds to CTCF sites at many distal regulatory elements identified by Cao et al. (2010). A third evidence is the fact that CTCF can also induce long-range chromatin interactions that culminate in silencing of genes important for muscle differentiation. This is illustrated by the gene *p57* whose product, a cdk inhibitor important for many cellular processes, and that has been shown deficient in cancer and other developmental disorders (Pateras et al., 2009).

Mechanistically, the imprinting control regulatory region KvDMR1, located around 150 kb away of the p57 transcriptional start site, contacts p57 promoter region in a CTCF-Rad21 dependent manner (Battistelli et al., 2014). As myogenic differentiation proceeds, MyoD binds to the KvDMR1 region, inducing the progressive loss of Rad21. Interestingly, CTCF remains at the sites of interactions, meaning that the locus is primed for looping and responsive to either MyoD or Rad21 (Busanello et al., 2012; Battistelli et al., 2014). These examples suggest that CTCF might have a crucial role during myogenic differentiation by establishing long-range chromatin interactions important in delimitating and constraining genes for expression at defined times of myogenesis (Battistelli et al., 2014).

### MyoD and the Three-Dimensional Organization of Chromatin

**Figure 2:**
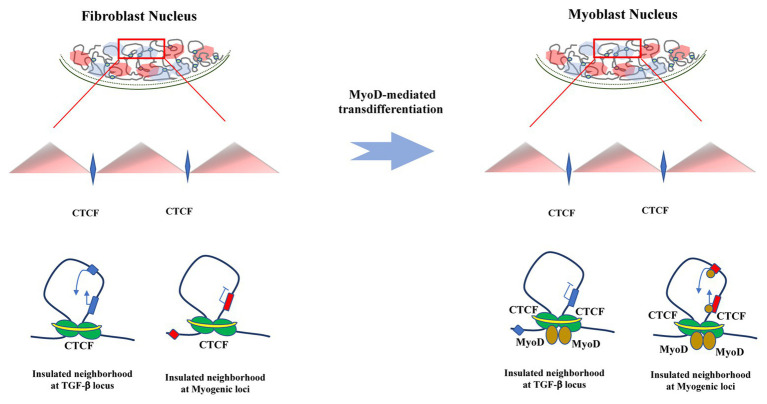
MyoD dependent trans-differentiation drives changes in chromatin interaction. Schematic representation of chromatin changes that MyoD drives during somatic reprogramming toward trans-differentiation. While MyoD erases the cell of origin transcriptional program by altering insulated neighborhoods that allow – among many others – TGF-β promoter-enhancer contacts in fibroblasts, it also activates skeletal myogenesis through reconfiguration of chromatin interactions that involves *cis*-regulatory and structural genomic elements and temporally precedes transcriptional regulation of muscle genes.

Additional experimental efforts showing MyoD-regulated chromatin interactions suggest that MyoD could regulate gene expression also by altering the three-dimensional genome architecture (Figure 2; Busanello et al., 2012; Battistelli et al., 2014; Harada et al., 2015). For instance, a 3C– and FISH-based study showed that a group of genes meant to be expressed at late times of differentiation are in close physical proximity, even when they are located at different chromosomes, and that share a repressed transcriptional state. However, interactions between these late genes with early expressed genes such as myogenin were not detected. The authors proposed a mechanism by which the formation of such interactions is dependent on the presence of MyoD and its association with HDAC1and the SWI/SNF ATPase, Brg-1 at poised myogenic genes (Harada et al., 2015). However, a plausible explanation of how a chromatin remodeling enzyme contributes to overall genome organization remains elusive and incomplete.

A more recent study took advantage of two biological properties of MyoD: (1) the ability that MyoD possess to virtually reprogram all somatic cells into skeletal muscles after ectopic expression and (2) the fact that MyoD-mediated trans-differentiation also permits the study of two separate and sequential stages of trans-differentiation: lineage commitment and terminal differentiation (Davis et al., 1987; Weintraub et al., 1989). This implies that MyoD possesses properties that enable epigenetic and transcriptional events necessary to coordinate repression of cell-of-origin gene expression and the transcription of new lineage-specific genes. In their study, Dall’Agnese et al. (2019) introduced an inducible MyoD transgene into human primary fibroblasts and interrogated by ChIP-seq whether it regulates gene expression by direct DNA binding. Among their findings, they report that MyoD binds to nearly 50,000 sites in myoblasts and 80,000 sites in differentiated myotubes. Importantly, only 5% of these MyoD binding sites were located at promoters of differentially expressed genes during differentiation. In addition to promoter elements, MyoD binding was detected at CTCF-binding sites and H3K27ac regions in both myoblasts and myotubes (Dall’Agnese et al., 2019). Importantly, upon MyoD expression in fibroblasts, inhibition of the original transcriptional program was observed, similar to what is seen in fibroblast reprogramming to induced-pluripotent stem cells (iPSCs) by over-expression of OCT4, SOX2, and NANOG (Ciglar et al., 2014; Chronis et al., 2017). These results indicate that master transcription factors share the ability to coordinately activate and repress specific transcriptional programs during reprogramming (Ciglar et al., 2014).

Further, *in situ* Hi-C (Rao et al., 2014) experiments revealed a pattern of co-regulation of genes within MyoD-bound topologically associated domains (TADs), where the 14% of the genome interacts in *-cis* within these elements during MyoD-dependent myogenic commitment and differentiation. In fact, the authors found a significant enrichment of MyoD binding at chromatin interactions involving promoter-promoter and promoter-enhancers pairs, indicating that MyoD is able to rewire chromatin architecture at promoter, enhancers, and insulators during fibroblast trans-differentiation into skeletal muscle. This MyoD-directed reconfiguration of chromatin interactions largely occurs at the subTAD level, by altering the structure of insulated neighborhoods, *via* binding at CTCF-anchored boundaries, as well as by targeting interactions inside insulated neighborhoods. Insulated neighborhoods, which are regions of the DNA that contain one or more genes and whose boundaries are co-bound by CTCF and cohesin, are important constituents of TADs or subTADs (Hnisz et al., 2013; Dowen et al., 2014; Ji et al., 2015; Narendra et al., 2015; Flavahan et al., 2016). Insulated neighborhoods also constrain gene regulation within their boundaries, by harboring interactions between *cis*-regulatory elements, such as promoter-enhancer communication (Sun et al., 2019). Since higher genomic structures such as TADs appear to be generally conserved, the fact that chromatin interactions within insulated neighborhoods could rather be cell-type-specific and dynamic (Dixon et al., 2015; Javierre et al., 2016; Bonev et al., 2017; Phanstiel et al., 2017; Siersbæk et al., 2017) is then relevant to note that MyoD is able to reconfigure insulated neighborhoods as nearly as 90% of its interaction sites with CTCF result higher at insulated neighborhoods boundaries (Dall’Agnese et al., 2019).

As MyoD is able to reconfigure chromatin in order to activate myogenic gene expression, it is also capable to repress inhibitors of muscle differentiation (Dall’Agnese et al., 2019). For example, TGF-β is a negative regulator of muscle differentiation (Liu et al., 2001; Hernández-Hernández et al., 2009) and is active in fibroblast. Importantly, its promoter was observed to interact with high frequency with its cognate enhancer in fibroblasts. Interestingly, this locus is contained within an insulator neighborhood whose boundaries are bound by MyoD in myoblasts after trans-differentiation. After MyoD introduction, interactions between these boundaries decreased along with TGF-β expression levels. On the contrary, upon MyoD expression in fibroblasts, increasing levels of the muscle specific genes *ITGA7* and *RDH5* were detected as well as binding of CTCF and MyoD at ITGA7 and RDH5 promoters. These observations showed that steady expression of MyoD is required for the maintenance of the three-dimensional chromatin landscape in order achieve myogenic commitment and differentiation (Dall’Agnese et al., 2019).

### Higher-Order Chromatin Organization and Muscle Disease

Chromosomal translocations causing gene fusions between FKHR (Foxo1) and Pax3 or Pax7 are characteristic of alveolar rhabdomyosarcoma (ARMS), a pediatric soft tissue cancer derived from the muscle lineage (Douglass et al., 1987). The translocation events fuse the transactivation domain of FHKR to the DNA binding domain of Pax3 or Pax7, leading to increased transcription from Pax3 or Pax7 response elements (Galili et al., 1993; Bennicelli et al., 1996; Barr, 2001). These chimeric proteins are expressed at high levels in ARMS tumors. Histologically, the tumors contain collections of poorly differentiated tissue, and weak evidence of muscle differentiation as marked by scant MyoD and desmin staining. Studies on the transcriptional behavior of Pax3-FKHR and Pax7-FKHR suggest that the chromosomal translocations exaggerate the normal function of Pax3 and Pax7 in myogenic progenitor cells, leading to dysregulation of growth, apoptosis, differentiation, and motility (Galili et al., 1993; Bennicelli et al., 1996; Barr, 2001).

The relevance of genomic translocations and rearrangements affecting how TADs organize is that they also alter networks of gene regulation relevant for the correct execution of many developmental programs (Li et al., 2018). In addition to its implication in Rhabdomyosarcoma, misregulation of Pax3 is also related with limb malformations. This occurs when deletions of complete parts of TADs and their telomeric boundaries promotes interactions between the enhancer element of the otherwise repressed gene *Epha4*, with Pax3. The resulting effect of Pax3 over-expression is a brachydactyly phenotype in mutant mice models (Lupiáñez et al., 2015). In the muscular context, the fusion of *Pax3* and *FKHR* genes associated with ARMS, promotes interaction of their regulatory elements and also generates a new TAD (Vicente-García et al., 2017). Finally, more comprehensive and detailed studies are needed in order to dissect the global effect of Pax3/7-FKHR fusions on the pathophysiology of Rhabdomyosarcomas.

## Concluding Remarks

Despite our current knowledge about the molecular and epigenetic mechanisms of myogenic commitment and differentiation, there is still a lack of precise information of how distal regulatory elements operate in the context of three-dimensional chromatin organization. Emerging studies and strategies are shedding light into these questions by the use of trans-differentiation cultures as well as primary cells. However, interrogating these aspects of genome regulation in freshly isolated muscle stem cells will be necessary in the attempt to translate new knowledge into regenerative medicine strategies. Although experimentally challenging, there are emerging attempts to perform genome-wide studies on global gene expression by single-cell RNA sequencing and chromatin accessibility assays by ATAC-seq. Perhaps, it is only a matter of time to capture the *in vivo* picture of how skeletal muscle commitment, differentiation, and regeneration are regulated in health and diseases.

Despite the advances in our understanding of key cellular processes mediating muscle regeneration at the molecular and epigenetic levels, translating these into therapeutic practices is still limited. Epigenetic modulators such as HDAC inhibitors have been used to promote regeneration and to reduce fibrosis in muscular dystrophies. However, a more precise and direct strategy is needed. Without the study and the complete understanding of heterogeneity of muscle stem cells and their relationship with niche-specific resident cells in homeostatic and regenerative contexts, we will be facing limited results in our attempt to tackle today’s most devastating muscle diseases. Single-cell transcriptomic analysis along with metabolome, proteome and epigenome information are only a part of the integrative approach that is until recently, being incorporated into experimental programs with the aim of more comprehensively understand the mechanisms of muscle regeneration and to design more effective therapeutics.

## Author Contributions

OH-H, RA-A, and JH-H researched the literature and wrote the manuscript. All authors contributed to the article and approved the submitted version.

### Conflict of Interest

The authors declare that the research was conducted in the absence of any commercial or financial relationships that could be construed as a potential conflict of interest.
